# Time variations in the transmissibility of pandemic influenza in Prussia, Germany, from 1918–19

**DOI:** 10.1186/1742-4682-4-20

**Published:** 2007-06-04

**Authors:** Hiroshi Nishiura

**Affiliations:** 1 Department of Medical Biometry, University of Tübingen, Westbahnhofstr. 55, Tübingen, D-72070, Germany; 2 Research Center for Tropical Infectious Diseases, Nagasaki University Institute of Tropical Medicine, 1-12-4 Sakamoto, Nagasaki, 852-8523, Japan

## Abstract

**Background:**

Time variations in transmission potential have rarely been examined with regard to pandemic influenza. This paper reanalyzes the temporal distribution of pandemic influenza in Prussia, Germany, from 1918–19 using the daily numbers of deaths, which totaled 8911 from 29 September 1918 to 1 February 1919, and the distribution of the time delay from onset to death in order to estimate the effective reproduction number, Rt, defined as the actual average number of secondary cases per primary case at a given time.

**Results:**

A discrete-time branching process was applied to back-calculated incidence data, assuming three different serial intervals (i.e. 1, 3 and 5 days). The estimated reproduction numbers exhibited a clear association between the estimates and choice of serial interval; i.e. the longer the assumed serial interval, the higher the reproduction number. Moreover, the estimated reproduction numbers did not decline monotonically with time, indicating that the patterns of secondary transmission varied with time. These tendencies are consistent with the differences in estimates of the reproduction number of pandemic influenza in recent studies; high estimates probably originate from a long serial interval and a model assumption about transmission rate that takes no account of time variation and is applied to the entire epidemic curve.

**Conclusion:**

The present findings suggest that in order to offer robust assessments it is critically important to clarify in detail the natural history of a disease (e.g. including the serial interval) as well as heterogeneous patterns of transmission. In addition, given that human contact behavior probably influences transmissibility, individual countermeasures (e.g. household quarantine and mask-wearing) need to be explored to construct effective non-pharmaceutical interventions.

## Background

In the history of human influenza, Spanish flu (1918–20), caused by influenza A virus (H1N1), has resulted in the biggest disaster to date. The disease is believed to have killed 20–100 million individuals worldwide, having a considerable impact on public health not only in the past but also in the present [1]. Although the detailed mechanisms of its pathogenesis have yet to be clarified, pandemic influenza is characterized by severe pulmonary pathology due to the highly virulent nature of the viral strain and the host immune response against it [2]. Even though future pandemic strains could potentially be different from that of Spanish flu, the threat of recent avian influenza epidemics is causing widespread public concern. In order to plan effective countermeasures against a probable future pandemic, a comprehensive understanding of the epidemiology of Spanish flu is crucial in offering insight into control strategies and clarifying what and how we should prepare for such an event at the community and individual level. Nevertheless, various epidemiological questions regarding the 1918–20 pandemic remain to be answered [3].

One use of historical epidemiological data is in quantification of the transmission potential of a pandemic strain, which can help determine the intensity of interventions required to control an epidemic. The most important summary measure of transmission potential is the basic reproduction number, *R*_0_, defined as the average number of secondary cases arising from the introduction of a single primary case into an otherwise fully susceptible population [4]. For example, one of the best known uses of *R*_0 _is in determining the critical coverage of immunization required to eradicate a disease in a randomly mixing population, *p*_c_, which can be derived using *R*_0_: *p*_c _> 1-1/*R*_0 _[5]. Moreover, knowing the *R*_0 _is a prerequisite for designing public health measures against a potential pandemic using simulation techniques. To date, the *R*_0 _of Spanish flu has been estimated using epidemiological records in the UK [6,7], USA [8-10], Switzerland [11], Brazil [12] and New Zealand [13], all of which suggested slightly different estimates. Whereas studies in the US and UK proposed an *R*_0 _ranging from 1.5–2.0 [6,7,9], other studies indicated that it could be closer to or greater than 3 [8,10-13]. In addition, an ecological modeling study proposed that the *R*_0 _of seasonal influenza is in the order of 20 [14], generating a great deal of controversy in its interpretation.

Another problem with Spanish flu data is that only a few studies have assessed the time course of the pandemic. Although effective interventions against influenza may have been limited in the early 20th century, it is plausible that the contact frequency leading to infection varied considerably with time owing to the huge number of deaths and dissemination of information through local media (e.g. newspapers and posters). To shed light on this issue, it is important to evaluate time-dependent variations in the transmission potential. Explanation of the time course of an epidemic can be partly achieved by estimating the effective reproduction number, *R*(*t*), defined as the actual average number of secondary cases per primary case at time *t *(for *t *> 0) [15-17]. *R*(*t*) shows time-dependent variation with a decline in susceptible individuals (intrinsic factors) and with the implementation of control measures (extrinsic factors). If *R*(*t*) < 1, it suggests that the epidemic is in decline and may be regarded as being 'under control' at time *t *(vice versa, if *R*(*t*) > 1).

This paper has two main purposes, the first of which is to examine one of the possible factors yielding the slightly different *R*_0_ estimates of pandemic influenza in recent studies. Specifically, this variation is examined in relation to the choice of a key model parameter (the serial interval) frequently derived from the literature. The second is to assess the transmissibility of pandemic influenza with time. The time course of a pandemic is likely to be influenced by heterogeneous patterns of transmission and human factors that modify the frequency of infectious contact with time. The latter aim is concerned with a common assumption in many influenza models, that the transmission rate is independent of time. Under this assumption, in a homogeneously mixing population, transmissibility with time has to be characterized only by the depletion of susceptible individuals due to infection, resulting in a monotonic decrease. However, this might not be true for Spanish flu, even though its social background (e.g. media reports and global alert) was rather different from that of severe acute respiratory syndrome (SARS) in 2002–03, for example, which accompanied huge behavioral changes. The daily number of deaths during the fall wave (from September 1918 – February 1919) and the relevant statistics in Prussia, Germany [18] (see [Additional file 1]), are used in the following analysis.

## Results

### Temporal distribution of influenza

**Figure 1:**
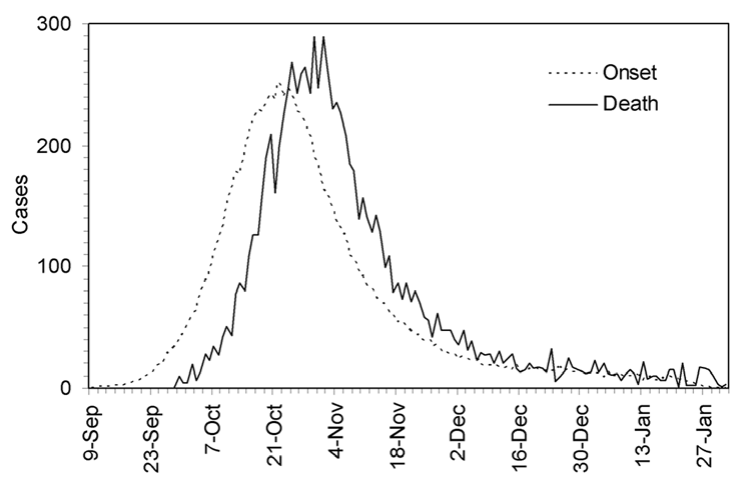
**Epidemic curve of pandemic influenza in Prussia, Germany, from 1918–19**. Reported daily number of influenza deaths (solid line) and the back-calculated temporal distribution of onset cases (dashed line). Daily counts of onset cases were obtained using the time delay distribution from onset to death (see Table 1). Data source: ref [18] (see [Additional file 1]).

**Figure 2:**
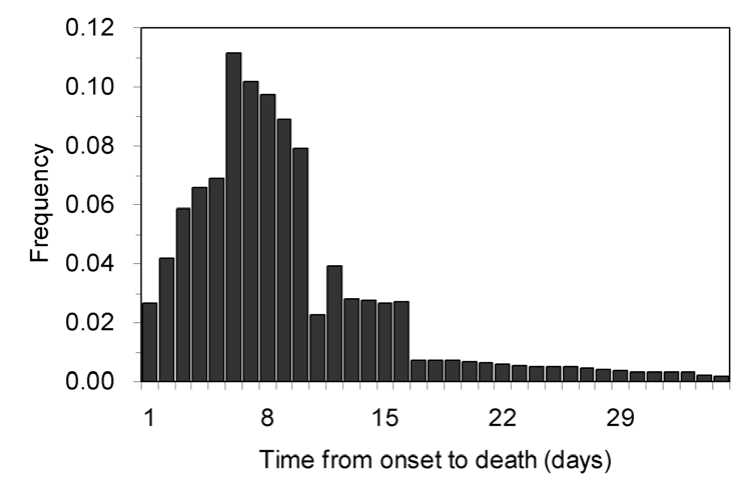
**Distribution of the time delay from onset to death during the influenza epidemic in Prussia, Germany, from 1918–19**. Time from disease onset (i.e. fever) to death is given for 6233 influenza deaths. A simple 5-day moving average was applied to the original data. Data source: ref [18] (see [Additional file 2]).

The daily number of influenza deaths from 29 September 1918 to 1 February 1919 was used in the following analyses (Figure 1) [18]. First, the temporal distribution of influenza deaths was transformed to the daily incidence (i.e. the daily case onset) using the time delay distribution from onset to death given in the same records. Figure 2 shows the time delay distribution, *f*(*τ*), the frequency of death *τ *days after onset (see [Additional file 2] for the original data). Assuming that the maximum time-lag from onset to death was 35 days, the mean (median and standard deviation) time delay would have been 9.0 (8.0 and 6.0) days, which is consistent with relevant data obtained in the US [8]. Figure 1 also shows the back-calculated distribution of the daily incidence, *C*(*t*), at time *t *(dashed line). The daily count of onset is most likely to have peaked on 22 October 1918 (Day 43), preceding the peak of influenza death by 8–10 days.

### Time variations in the transmission potential

**Figure 3:**
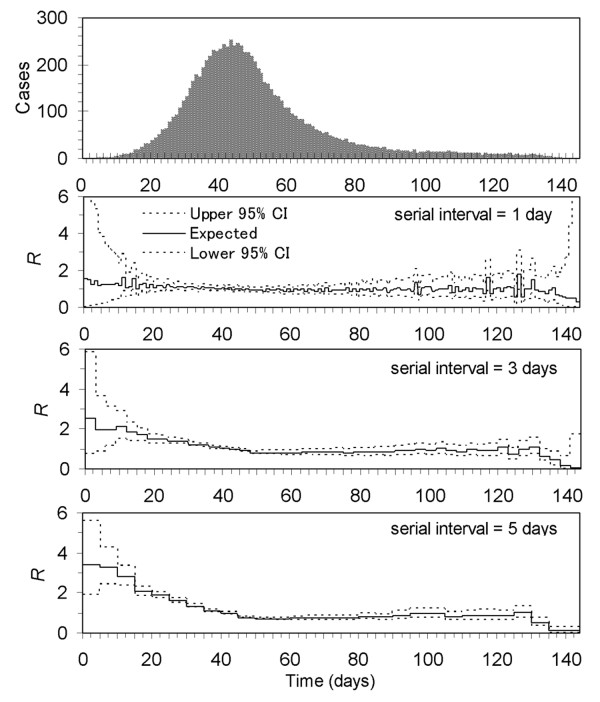
**Epidemic curve and the corresponding effective reproduction numbers (*R*) with variable serial intervals**. Time variation in the effective reproduction number (the number of secondary infections generated per case by generation) assuming three different serial intervals is shown. The serial interval was assumed to be 1 (second from the top), 3 (lower middle) and 5 days (bottom). Days are counted from September 9, 1918, onwards.

Next, time-inhomogeneous evaluation was performed, focusing on the serial interval, the time between infection of one person and infection of others by this individual (or the time from symptom onset in an index case to symptom onset in secondary cases) [19,20]. Figure 3 shows time variations in the estimated effective reproduction numbers obtained assuming three different serial intervals (i.e. 1, 3 and 5 days) compared with the corresponding epidemic curve. Epidemic date 0 represents 9 September 1918 when the back-calculated onset of cases initially yielded a value the nearest integer of which was 1. Since the precision of the estimate is influenced by the observed number of cases, wide 95% confidence intervals (CI) were observed for estimates using a short serial interval. However, these time variations in *R*(*t*) exhibited similar qualitative patterns: (i) although the *R*(*t*) was highest at the beginning of the epidemic, the estimates fell below 1 when the epidemic curve came close to the peak (i.e. Days 45–50). For example, the estimated *R*(*t*) at Day 50 was 0.92 (95% CI: 0.79, 1.06), 0.82 (0.75, 0.89) and 0.72 (0.67, 0.78), respectively, for a serial interval of 1, 3 and 5 days. This period corresponds to the time when public health measures were instituted, e.g. obligatory case reporting, encouragement of mask wearing, and closing of public buildings such as churches and theaters [18,21]. (ii) Thereafter, *R*(*t*) stayed slightly below unity, reflecting a slow decline in the number of onset cases. (iii) Shortly before the end of the epidemic (i.e. Days 90–120), *R*(*t*) increased again above 1. (iv) Finally, the expected values of *R*(*t*) fell below 1 very close to the end of the epidemic. In this stage, estimates assuming a short serial interval exhibited wide uncertainty bounds, reflecting stochasticity due to the small number of cases.

### Estimates of R and the serial interval

**Figure 4:**
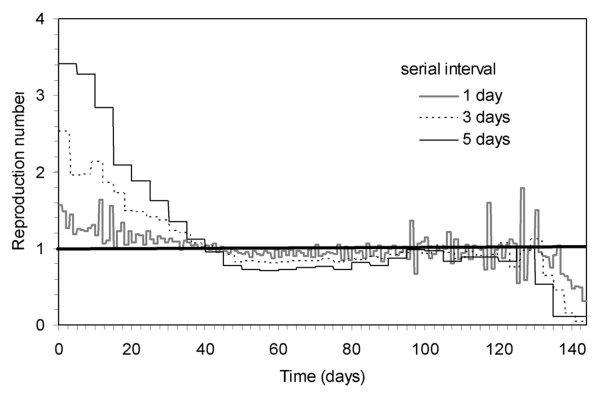
**Comparison of the effective reproduction number assuming different serial intervals**. Expected values of the effective reproduction number with a serial interval of 1 (grey), 3 (dashed black) and 5 days (solid black). The horizontal solid line represents the threshold value, *R *= 1, below which the epidemic will decline to extinction. Days are counted from September 9, 1918, onwards.

Figure 4 compares the expected values of *R*(*t*) assuming each of the serial intervals employed. Although the possibility of individual heterogeneity (e.g. potential superspreaders in the early stage) cannot be excluded [22], *R*(*t*) at time *t *= 0 is theoretically equivalent to *R*_0_. Assuming serial intervals of 1, 3 and 5 days, *R*_0 _was estimated to be 1.58 (95% CI: 0.03, 10.32), 2.52 (0.75, 5.85) and 3.41 (1.91, 5.57), respectively. It is remarkable, therefore, to see that *R*(*t*) largely depends on the assumed length of the serial interval. That is, the longer the serial interval, the higher the *R*(*t*). It should also be noted that the relationship between *R*(*t*) and the serial interval is reversed when the epidemic is under control (i.e. when *R*(*t*) < 1 in the later stage of the epidemic).

Table 1 shows recently reported estimates of *R*_0 _during the fall wave of Spanish flu according to the estimated magnitude of transmissibility. Although two studies (in the UK [7] and New Zealand [13]; which appear in bold in the table) were based on model assumptions and a specific setting different from those in other countries (this point is discussed below), there are two tendencies that are consistent with the findings of the present study. The first is the relationship between *R*_0 _and the serial interval described above. The reported estimates of *R*_0 _roughly correspond to the assumed length of the serial interval, estimates of which are frequently derived from the literature. Although the New Zealand study differs in that the estimates were obtained from close contact data in an army camp, the above-described relationship was also the case for the three different estimates. The second tendency shown in Table 1 relates to the estimates of *R*_0 _obtained by fitting the model to the entire epidemic curve without taking time variations into account (referred to as an autonomous system), thus tending to yield high estimates. Fitting such a model to the entire epidemic curve will probably lead to overestimations of *R*_0 _as time variations in secondary transmissions are ignored.

**Table 1:** Reported estimates of the basic reproduction number of pandemic influenza during the fall wave (2nd wave) from 1918–19

Location	Serial interval (days)	*R*_0_	Fitting of a time-independent system with the entire epidemic curve	Reference
San Francisco, USA	6 6	3.5 2.4	Yes No	10
45 cities in the USA	6^†^	2.7	No	8
**UK (entire England and Wales)**^‡^	**6**	**1.6**	**Yes**	**7**
Geneva, Switzerland	5.7	3.8	Yes	11
Sao Paulo, Brazil	4.6^§^	2.7	Yes	12
83 cities in the UK	3.2 and 2.6	1.7–2.0	No	6
45 cities in the USA	2.9	1.7	No	9
**Featherston Military Camp, New Zealand**^¶^	**1.6 ** **1.1 ** **0.9**	**3.1 ** **1.8 ** **1.3**	**Yes**	**13**

^†^Sensitivity of the *R *estimates to different assumptions for the serial interval was examined; ^‡^Three pandemic waves were simultaneously fitted assuming a large number of susceptible individuals but the statistical details were not given;^§^Parameter estimates from previous work were shown [40], but these did not match the assumed parameter values given in the Table; ^¶^The epidemic was observed in a community with closed contact (i.e. military camp).

### Simulated epidemic curve

**Figure 5:**
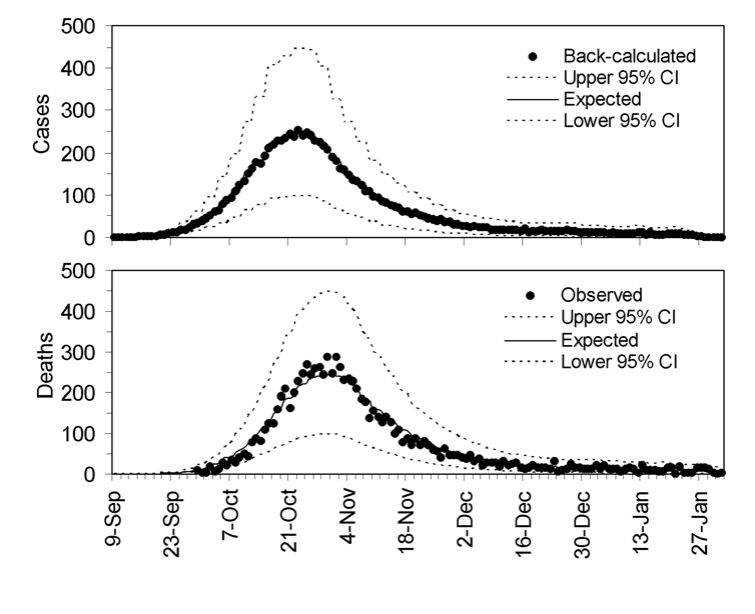
**Simulated epidemic curve of pandemic influenza in Prussia, Germany, from 1918–19**. Comparison of observed epidemic curves of onset (top) and death (bottom) with simulated curves. Expected values of influenza cases and deaths (solid line) mainly overlapped with the observed numbers (dot). Dashed lines indicate the corresponding upper and lower 95% confidence intervals (CI) based on 1000 simulation runs. The 95% CI of cases and deaths were determined by 2.5th and 97.5th percentiles of the simulated cases and deaths at each time point.

Stochastic simulations were performed to assess the performance of the proposed model. Figure 5 compares the simulated numbers of cases and deaths, assuming a serial interval of 3 days, with the observed epidemic. By definition (i.e. using equation (3); see Methods), the expected values of cases and deaths obtained using the estimated *R*(*t*) reflected the observed epidemic curves reasonably. On the basis of 1000 simulation runs, the mean epidemic size was 8911 deaths (95% CI: 3375, 16240). Within this range, the epidemic varied widely in size. Of the total number of simulations, 948 declined to extinction within the observed time period (i.e. before 1 February 1919). The highest frequency of extinction (n = 486 runs, 51.3%) was observed in the last interval (i.e. the 48th interval since the beginning of the epidemic). The mean and median (25 to 75% quartile) times of extinction were 140.9 and 144 (141 to 144) epidemic days, respectively. The simulation results obtained assuming serial intervals of 1 and 5 days also reflected the observed epidemic curve reasonably (data not shown), with wide 95% CI in the simulations using a short serial interval.

## Discussion

This paper has examined time variations in the transmission potential of pandemic influenza in Prussia, Germany, from 1918–19. *R*(*t*) was estimated using a discrete-time branching process, allowing reasonable assessment of the impact of the serial interval. Whereas two different stochastic models have been proposed to quantify the time variations in transmission rate [23,24], the present study showed that reasonable estimates of *R*(*t*) can be inferred using a far simpler method without assuming the number of susceptible individuals or further details of the disease dynamics. There were two important findings. First, *R*(*t*) depends on the assumed length of the serial interval; second, it varied with time and did not decline monotonically, reflecting underlying time variations in secondary transmission. In the Prussian epidemic, *R*(*t*) stayed close to 1 in the middle of the epidemic and then increased at a later stage.

In addition, the different recently reported *R*_0 _estimates for pandemic influenza were implicitly compared. Long serial intervals, estimates of which are often derived from the literature, seem to have yielded high estimates of *R*_0_, the relationship of which has been extensively investigated in previous studies by means of sensitivity analysis [8,25], implying that a precise estimate of the serial interval is crucial for elucidating the finer details of *R*_0 _[9]. This point has to be interpreted cautiously in relation to Table 1, since essentially there are two potential sources of variations in *R*_0_:

(A) Estimates of *R*_0 _will greatly vary according to model assumptions and the structure and type of data used to infer the relevant parameters [26].

(B) *R*_0 _can differ with time and place. That is, the transmission potential is generally influenced by various underlying social and biological conditions (e.g. contact patterns, differential susceptibility and pathogenic factors) [27,28].

It should be noted that the present study examined only some of the factors related to (A) and did not explicitly test this hypothesis. Indeed, there are other plausible explanations for the variations in *R*_0 _in Table 1. For example, point (A) may be particularly true for the UK study, the small estimates of which may be attributable to the modeling assumption that fitted the model to three waves of the pandemic [7]. Moreover, the New Zealand study is a good example of point (B) [13]. This epidemic was observed in a community with closed contact (i.e. an army camp), which could result in high estimates of *R*_0 _even assuming a short serial interval. Thus, no definitive reason for the differences in *R*_0 _can be clarified unless each model is examined in relation to others, permitting explicit comparisons and robustness assessment [26]. However, despite this, it is remarkable that differences in *R*(*t*) were obtained using the assumed serial interval lengths employed in the present study and that the differences in the *R*_0 _of pandemic influenza were also consistent with this well-known relationship (i.e. between *R*_0 _and the serial interval). The finding implies that it is critically important to clarify details of the natural history of a disease in order to offer robust assessments. In addition, further controversy concerning the *R*_0 _of seasonal influenza (= 20) needs to be addressed by exploring in detail the immune protection mechanisms of influenza [14].

The second finding of the present study concerns the time variations in secondary transmission. Although it is commonly assumed that a large epidemic only declines to extinction with depletion of susceptible individuals, this assumption leads to a monotonic decline in *R*(*t*). That is, in a homogeneously mixing population, *R*(*t*) is given by *R*_0_*S*(*t*)/*S*(0), where *S*(*t*) is the number of susceptible individuals at time *t *[29]. Whereas the decline in *R*(*t*) in Prussia probably reflected a decline in susceptible individuals, the observed qualitative pattern (i.e. a non-monotonic decline in *R*(*t*)) is likely to have involved other factors not included in usual assumptions of homogeneously mixing models. The non-monotonic decline in *R*(*t*) could reflect (i) heterogeneous patterns of transmission and/or (ii) other time-dependent underlying factors. For example, two important factors need to be discussed with regard to heterogeneous transmission. The first, age-related heterogeneity in transmission was ignored in the present study. Whereas the case fatality of pandemic influenza varied with age (exhibiting a W-shaped curve not only for mortality but also for case fatality [3]), the present study assumed fixed and crude case fatality for the entire population. Thus, if the age-related transmission patterns yield time variations in age-specific incidence [30], the decline in *R*(*t*) could partly be attributable to age-related heterogeneity. Similarly, the time from onset to death may also vary by age-related factors. The second important factor is social heterogeneity in transmission (e.g. spatial spreading patterns). For example, considering realistic patterns of influenza spread in a location with urban and rural sub-regions, slow decline in incidence could originate from heterogeneous spatial spread between and within rural sub-regions. If some rural areas previously free from influenza are infested by a few cases at some point in time, such local spread could modify the overall epidemic curve. Since the present study assumed a closed population because detailed data were lacking, additional information (e.g. cases with time and place) is needed to elucidate the finer details.

With respect to (ii), other time-dependent underlying factors, it is likely that public health measures as well as human contact behaviors (including human migration) also influence the time course of an epidemic. From a very early study [31], it has been suggested that human behavioral changes (or differing transmission rates due to time-varying contact patterns) are observed during the course of an epidemic. If this is the case, the finding suggests that time-varying transmission potential is not only the case for SARS (i.e. recent epidemics accompanied by considerable media coverage) [15,32,33] but also for historical epidemics with a huge magnitude of disaster. Indeed, recent studies on Spanish flu in the US that employed rough assumptions implied that interventions had a considerable impact on the time trend [34,35]. This also reasonably explains why high estimates of *R*_0 _are likely to originate from fitting an autonomous model to the entire epidemic curve. In practical terms, such a result implies that human behaviors could considerably influence transmissibility, and moreover, could potentially be a necessary countermeasure. Understanding the significant impact of human contact behaviors on the time course is therefore of importance [31]. For example, non-pharmaceutical individual countermeasures are crucial for poor resource settings, especially in developing countries [36]. In addition to community-based measures such as social distancing and area quarantine, it is also crucial to suggest what can be done at the individual level. In line with this, the effectiveness of individual countermeasures (e.g. household quarantine and mask wearing) needs to be further explored using additional data (i.e. of seasonal influenza) and models.

## Conclusion

In summary, this paper showed the relationship between the *R*(*t*) and serial interval and assessed time variations in the transmissibility of pandemic influenza. The findings imply a need to detail the natural history of influenza as well as heterogeneous patterns of transmission, suggesting that robust assessment can only be made when population- and individual-based disease characteristics are clarified [37] and implying that further observations in clinical and public health practice are crucial. Given that individual human contact behaviors could influence the time variations in transmission potential, further understanding of the importance of individual-based countermeasures (e.g. household quarantine and mask wearing) could therefore offer hope for development of effective non-pharmaceutical interventions.

## Methods

### Data

Medical officers in Prussia recorded the daily number of influenza deaths from 29 September 1918 to 1 February 1919 (Figure 1) [18]; a total of 8911 deaths were reported (see [Additional file 1]). Throughout the pandemic period in Germany, the largest number of deaths was seen in this fall wave [21]. Prussia represents the northern part of present Germany and at the time of the pandemic was part of the Weimer Republic as a free state following World War I. The death data were collected from 28 different local districts surrounding the town of Arnsberg, which, at the time of the epidemic, had a population of approximately 2.5 million individuals (the mortality rate in this period being 0.36%). Although case fatality for the entire observation area was not documented, the numbers of cases and deaths during part of the fall wave were recorded for 25 districts. Among a total of 61,824 cases, 1609 deaths were observed, yielding a case fatality estimate of 2.60% (95% CI: 2.48, 2.73). For simplicity, the inflow of infected individuals migrating from other areas was ignored in the following analysis.

### Back-calculation of the daily case onset

The daily incidence (i.e. daily case onset) was back-calculated using the daily number of influenza deaths (Figure 1) and the time delay distribution from onset to death (Figure 2; also see [Additional file 2]). Given *f*(*τ*), the frequency of death *τ *days after onset, the relationship between the reported daily number of deaths, *D*(*t*), and daily incidence, *C*(*t*), at time *t *is given by:

D(t)=p∫0tC(t−τ)f(τ)dτ
 MathType@MTEF@5@5@+=feaafiart1ev1aaatCvAUfKttLearuWrP9MDH5MBPbIqV92AaeXatLxBI9gBaebbnrfifHhDYfgasaacH8akY=wiFfYdH8Gipec8Eeeu0xXdbba9frFj0=OqFfea0dXdd9vqai=hGuQ8kuc9pgc9s8qqaq=dirpe0xb9q8qiLsFr0=vr0=vr0dc8meaabaqaciaacaGaaeqabaqabeGadaaakeaacqWGebarcqGGOaakcqWG0baDcqGGPaqkcqGH9aqpcqWGWbaCdaWdXaqaaiabdoeadjabcIcaOiabdsha0jabgkHiTGGaciab=r8a0jabcMcaPiabdAgaMjabcIcaOiab=r8a0jabcMcaPiabdsgaKjab=r8a0bWcbaGaeGimaadabaGaemiDaqhaniabgUIiYdaaaa@469A@

where *p *is the case fatality ratio, which is independent of time. Although the case fatality, *p*, was not taken into account in Figure 1, the following model reasonably cancels out the effect of *p *assuming that the conditional probability of death given infection is independent of time.

### Estimation of the reproduction number

The effective reproduction number at time *t*, *R*(*t*), can be back-calculated using the incidence, *C*(*t*), and serial interval distribution, *g*(*τ*), of length *τ *:

C(t)=∫0tC(t−τ)R(t−τ)g(τ)dτ
 MathType@MTEF@5@5@+=feaafiart1ev1aaatCvAUfKttLearuWrP9MDH5MBPbIqV92AaeXatLxBI9gBaebbnrfifHhDYfgasaacH8akY=wiFfYdH8Gipec8Eeeu0xXdbba9frFj0=OqFfea0dXdd9vqai=hGuQ8kuc9pgc9s8qqaq=dirpe0xb9q8qiLsFr0=vr0=vr0dc8meaabaqaciaacaGaaeqabaqabeGadaaakeaacqWGdbWqcqGGOaakcqWG0baDcqGGPaqkcqGH9aqpdaWdXaqaaiabdoeadjabcIcaOiabdsha0jabgkHiTGGaciab=r8a0jabcMcaPiabdkfasjabcIcaOiabdsha0jabgkHiTiab=r8a0jabcMcaPiabdEgaNjabcIcaOiab=r8a0jabcMcaPiabdsgaKjab=r8a0bWcbaGaeGimaadabaGaemiDaqhaniabgUIiYdaaaa@4C2E@

Equation (2) is a slightly different expression of a method proposed for SARS [15]. The advantages of this model include: (i) we only need to know the time of onset of cases (i.e. the model does not require the total number of susceptible individuals or detailed contact information) and (ii) the time-dependent reproduction number can be reasonably estimated using a far simpler equation than other population dynamics models. Unfortunately, detailed information on the distribution of the serial interval, *g*(*τ*), is not available for pandemic influenza, and historical records often offer only an approximate mean length. Although a recent study estimated the serial interval from household transmission data of seasonal influenza [9], this is likely to have been considerably underestimated owing to the short interval from onset to secondary transmission within the households examined. Thus, the analyses conducted in the present study simplify the model using various mean lengths of the serial interval assumed in previous works. Supposing that we observed *C*_i _cases in generation *i*, the expected number of cases in generation *i*+1, *E*(*C*_i+1_) occurring a mean serial interval after onset of *C*_i _is given by:

*E*(*C*_*i *+ 1_) = *C*_*i*_*R*_*i*_

where *R*_i _is the effective reproduction number in generation *i*. That is, cases in each generation, *C*_1_, *C*_2_, *C*_3_, ..., *C*_n _are given by *C*_0_*R*_0_, *C*_1_*R*_1_, *C*_2_*R*_2_, ..., *C*_n-1_*R*_n-1 _and also by *C*_0_*R*_0_, *C*_0_*R*_0_*R*_1_, *C*_0_*R*_0_*R*_1_*R*_2_, ..., L=constant×∏j=0N−1(CjRj)Cj+1exp⁡(−CjRj)
 MathType@MTEF@5@5@+=feaafiart1ev1aaatCvAUfKttLearuWrP9MDH5MBPbIqV92AaeXatLxBI9gBaebbnrfifHhDYfgasaacH8akY=wiFfYdH8Gipec8Eeeu0xXdbba9frFj0=OqFfea0dXdd9vqai=hGuQ8kuc9pgc9s8qqaq=dirpe0xb9q8qiLsFr0=vr0=vr0dc8meaabaqaciaacaGaaeqabaqabeGadaaakeaacqWGmbatcqGH9aqpcqqGJbWycqqGVbWBcqqGUbGBcqqGZbWCcqqG0baDcqqGHbqycqqGUbGBcqqG0baDcqGHxdaTdaqeWbqaaiabcIcaOiabdoeadnaaBaaaleaacqWGQbGAaeqaaOGaemOuai1aaSbaaSqaaiabdQgaQbqabaGccqGGPaqkdaahaaWcbeqaaiabdoeadnaaBaaameaacqWGQbGAcqGHRaWkcqaIXaqmaeqaaaaakiGbcwgaLjabcIha4jabcchaWjabcIcaOiabgkHiTiabdoeadnaaBaaaleaacqWGQbGAaeqaaOGaemOuai1aaSbaaSqaaiabdQgaQbqabaGccqGGPaqkaSqaaiabdQgaQjabg2da9iabicdaWaqaaiabd6eaojabgkHiTiabigdaXaqdcqGHpis1aaaa@5C65@, respectively. By incorporating variations in the number of secondary transmissions generated by each case into the same generation (referred to as offspring distribution), the model can be formalized using a discrete-time branching process [38]. The Poisson process is conventionally assumed to model the offspring distribution, representing stochasticity (i.e. randomness) in the transmission process. This assumption indicates that the conditional distribution of the number of cases in generation *i*+1 given *C*_i _is given by:

*C*_*i *+ 1_|*C*_*i *_~ Poisson[*C*_*i*_*R*_*i*_]

For observation of cases from generation 0 to *N*, the likelihood of estimating *R*_i _is given by:

L=constant×∏j=0N−1(CjRj)Cj+1exp⁡(−CjRj)
 MathType@MTEF@5@5@+=feaafiart1ev1aaatCvAUfKttLearuWrP9MDH5MBPbIqV92AaeXatLxBI9gBaebbnrfifHhDYfgasaacH8akY=wiFfYdH8Gipec8Eeeu0xXdbba9frFj0=OqFfea0dXdd9vqai=hGuQ8kuc9pgc9s8qqaq=dirpe0xb9q8qiLsFr0=vr0=vr0dc8meaabaqaciaacaGaaeqabaqabeGadaaakeaacqWGmbatcqGH9aqpcqqGJbWycqqGVbWBcqqGUbGBcqqGZbWCcqqG0baDcqqGHbqycqqGUbGBcqqG0baDcqGHxdaTdaqeWbqaaiabcIcaOiabdoeadnaaBaaaleaacqWGQbGAaeqaaOGaemOuai1aaSbaaSqaaiabdQgaQbqabaGccqGGPaqkdaahaaWcbeqaaiabdoeadnaaBaaameaacqWGQbGAcqGHRaWkcqaIXaqmaeqaaaaakiGbcwgaLjabcIha4jabcchaWjabcIcaOiabgkHiTiabdoeadnaaBaaaleaacqWGQbGAaeqaaOGaemOuai1aaSbaaSqaaiabdQgaQbqabaGccqGGPaqkaSqaaiabdQgaQjabg2da9iabicdaWaqaaiabd6eaojabgkHiTiabigdaXaqdcqGHpis1aaaa@5C65@

Since the Poisson distribution represents a one parameter power series distribution, the expected values and uncertainty bounds of *R*_i _can be obtained for each generation. The 95% CI were derived from the profile likelihood. Since the length of the serial interval in previous studies ranged from 0.9 to 6 days [8,10,13], three different fixed-length serial intervals (i.e. 1, 3 and 5 days) were assumed for equation (5) with respect to the observed data. Although application of the Heaviside step function for the serial interval suffers some overlapping of cases in successive generations, this study ignored this and, rather, focused on the time variation in transmissibility using this simple assumption. For each length, the daily number of cases was grouped by the determined serial interval length. Whereas the choice of serial interval therefore affects estimates of *R*_i_, it does not affect the ability to predict the temporal distribution of cases. It should be noted that this simple model assumes a homogeneous pattern of spread.

### Stochastic simulation

To assess the performance of the above-described estimation procedure, stochastic simulations were conducted. The simulations directly used the branching process model, the offspring distribution of which follows a Poisson distribution with expected values, *R*_i_, estimated for each interval, *i*. Although the offspring distribution tends to exhibit a right-skewed shape (which was approximated by negative binomial distributions in recent studies [15,22,39]), it is difficult to extract additional information from the temporal distribution of cases only, so this paper focused on time variations in *R*(*t*) rather than individual heterogeneity. Each simulation was run with one index case at epidemic day 0. For the first two serial intervals, primary cases were set to generate 2.52 and 1.95 secondary cases deterministically in order to avoid immediate stochastic extinctions. Simulations were run 1000 times.

## Competing interests

The author(s) declare that they have no competing interests.

## Authors' contributions

HN carried out paper reviews, proposed the study, performed mathematical analyses and drafted the manuscript. The author has read and approved the final manuscript.

## Supplementary Material

Additional File 1 Reported daily number of influenza deaths in Prussia, Germany, from 1918–19. The temporal distribution of influenza deaths is given in Microsoft Excel format. Data source: ref. [18]. Click here for file

Additional File 2 Time delay from onset to death during the influenza epidemic in Prussia, Germany, from 1918–19. Data source: ref. [18]. Click here for file
